# Prestige Affects Cultural Learning in Chimpanzees

**DOI:** 10.1371/journal.pone.0010625

**Published:** 2010-05-19

**Authors:** Victoria Horner, Darby Proctor, Kristin E. Bonnie, Andrew Whiten, Frans B. M. de Waal

**Affiliations:** 1 Living Links, Yerkes National Primate Research Center, Emory University, Lawrenceville, Georgia, United States of America; 2 Department of Psychology, Beloit College, Beloit, Wisconsin, United States of America; 3 Centre for Social Learning and Cognitive Evolution, School of Psychology, University of St Andrews, Fife, Scotland, United Kingdom; Università di Parma, Italy

## Abstract

Humans follow the example of prestigious, high-status individuals much more readily than that of others, such as when we copy the behavior of village elders, community leaders, or celebrities. This tendency has been declared uniquely human, yet remains untested in other species. Experimental studies of animal learning have typically focused on the learning mechanism rather than on social issues, such as who learns from whom. The latter, however, is essential to understanding how habits spread. Here we report that when given opportunities to watch alternative solutions to a foraging problem performed by two different models of their own species, chimpanzees preferentially copy the method shown by the older, higher-ranking individual with a prior track-record of success. Since both solutions were equally difficult, shown an equal number of times by each model and resulted in equal rewards, we interpret this outcome as evidence that the preferred model in each of the two groups tested enjoyed a significant degree of prestige in terms of whose example other chimpanzees chose to follow. Such prestige-based cultural transmission is a phenomenon shared with our own species. If similar biases operate in wild animal populations, the adoption of culturally transmitted innovations may be significantly shaped by the characteristics of performers.

## Introduction

The impressive geographic variation in chimpanzee (*Pan troglodytes*) behavior is thought to be cultural in that it results from the transmission of socially acquired habits. Comparisons between African field sites, many of which have been in operation for several decades [1], [2], have revealed variations in dozens of courtship, communication, grooming, and tool-use behaviors, which differ between sites without obvious genetic or ecological explanations [3]–[5]. These findings raise questions about the evolution of our own cultural behavior and the extent to which chimpanzee and human cultures rely on the same social and cognitive processes. Like human culture, chimpanzee cultures likely arise when new behaviors are introduced to a population either through immigration by an outsider into an established group or by invention from within [6]–[9]. New behaviors may then be picked up by the rest of the group through social learning [10]. However, whether or not a new behavior is copied by others to become part of daily life likely depends on social variables such as the relationship of potential learners with the original model [10]–[13].

In nonhuman primates, as with human society, learning takes place in a structured social context [13], [14] and the nature of social relationships may directly influence who learns from whom [10], [15]. New learners have a choice of social models within a given group, but little is known about if, and how, they differentiate between these. Learners may be highly selective, using social cues such as model proficiency [16], dominance rank [17], age [18], or social affiliation [15], [19]–[21] to determine whom to copy. Consequently, cultural transmission may be impeded if new behaviors are introduced to a group by non-preferred social models [15], [22], [23]. However, such effects remain to be established experimentally.

With regards to human culture, great emphasis has been placed on the status or “prestige” of successful social models, such that individuals with previously demonstrated skills and knowledge earn respect and credibility, and their actions have a disproportionate influence on the behavior of others [24]. The role of prestige is sometimes presented as uniquely human [25] despite the absence of comparative research on this important topic.

Here, we set out to systematically investigate whether prestige effects might operate in the transmission of chimpanzee behaviors. We examined the role of social dynamics in learning by giving chimpanzees opportunities to learn different foraging behaviors from either of two conspecific models with different social characteristics. We tracked the transmission of each behavior to determine which model was copied most frequently. This procedure was repeated with two different chimpanzee groups (Group 1 and Group 2) such that chimpanzees in both groups observed a pair of trained group members performing each of the foraging behaviors.

## Materials and Methods

The experiment was conducted with two socially housed chimpanzee groups at the Yerkes National Primate Research Center's Field Station near Atlanta. The chimpanzees live in spacious outdoor enclosures (Group 1 = 711 m^2^; Group 2 = 528 m^2^) with grass, wooden climbing structures and enrichment toys. Each enclosure is attached to an indoor building with five interconnected bedrooms containing sleeping platforms, swings and nesting material. Both groups can hear, but not see each other because their enclosures are ∼200 m apart and separated by a small hill. Participation in research is voluntary. The chimpanzees recognize their names and can be ‘asked’ to participate in studies by calling them inside from the outside enclosures, or placing apparatus at the enclosure fence and giving them the choice to interact with it. All food rewards used in the study were supplemental to the chimpanzees' daily intake. In addition to daily meals, the chimpanzees receive behavioral enrichment in the form of foraging puzzles and novel objects. The procedures used in this study were entirely behavioral and lasted for no more than 20 min. All procedures were approved by Emory University's Institutional Animal Care and Use Committee. The Yerkes National Primate Research Center is accredited by the American Association for Accreditation of Laboratory Animal Care.

In each of the two groups, we selected a pair of female models (model A and model B) so that there were four models in total. In order to test the influence of social dynamics on learning, we maximized the potential social differences between the models, such that model A was older, held one of the highest social ranks in her group (based on daily observations) and had successfully introduced novel behaviors on several previous occasions [20], [26]–[28]. In contrast, model B was younger, held one of the lowest social ranks and had no previous experience in introducing novel behaviors.

**Figure 1:**
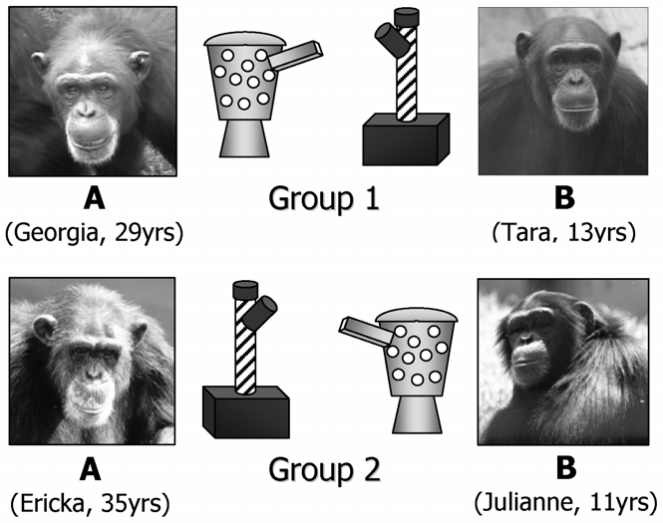
Model and apparatus allocations for chimpanzees in groups 1 and 2. Group 1 (top): Model A was trained to deposit tokens into the spotted receptacle while model B was trained to use the striped receptacle. Group 2 (bottom): Models A and B were trained to use the opposite receptacles from Group1.

In Group 1, model A was trained to collect plastic tokens and deposit them in a spotted receptacle attached to the fence of the outdoor enclosure, in order to receive food. Model B was trained to do the same for a striped receptacle 10 m from the first receptacle. In Group 2, the model and receptacle assignments were reversed (Fig. 1). Since both solutions were (i) equally difficult, (ii) demonstrated an equal number of times, (iii) resulted in an equal food reward, and (iv) counterbalanced between groups, any preference by observers to later prefer one receptacle over the other was likely influenced by the social characteristics of the model. We chose alternative solutions that were relatively unchallenging to chimpanzees to enable us to focus on the decision-making processes of the observers in terms of which model to copy. Depositing tokens is not a naturally occurring chimpanzee behavior. However, the social learning mechanisms required to learn this behavior sequence are likely to be similar to those used by chimpanzees in both captive and wild settings to learn a variety of behavior patterns via observation. Data from this study are therefore relevant to the behavior of wild chimpanzees and the potential transmission of chimpanzee cultures.

**Figure 2:**
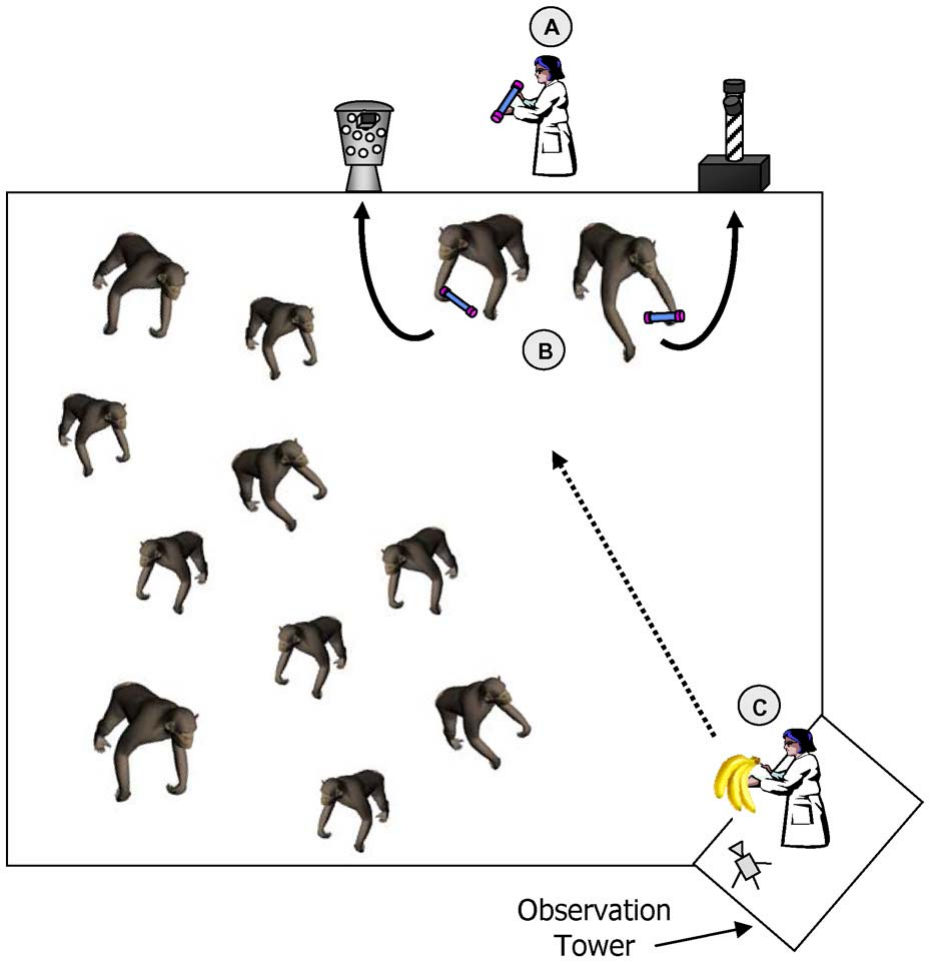
Experimental procedure used during the observation period. (A) trained models retrieve a token from an experimenter standing between the receptacles outside the enclosure fence; (B) models deposit their token into their respective receptacles; (C) a food reward is thrown to the model by a second experimenter standing on an observation tower.

In each group, before the experiment began the chimpanzees could observe both models A and B simultaneously perform their trained solution to the task during 20-minute sessions, with one session per day, conducted over 10 days (Fig. 2). In Group 1, both models performed 33 demonstrations and in Group 2, both models performed 46 demonstrations, so that the number of demonstrations performed by models A and B was equal *within* each group.

The critical test trials were conducted during three 20-minute sessions on separate days. At the start of each session, models A and B deposited one token into their trained receptacles. The task was then made available to all other chimpanzees but models were not given further tokens. Data collection was stopped on the third day because all chimpanzees who showed interest in the study had attempted the task. We were also concerned that future new performers would have a variety of social models to choose from, which could confound our ability to assess the relative influence of the original trained models. Further details about the materials and methods can be found in Supporting Information S1.

## Results

**Figure 3:**
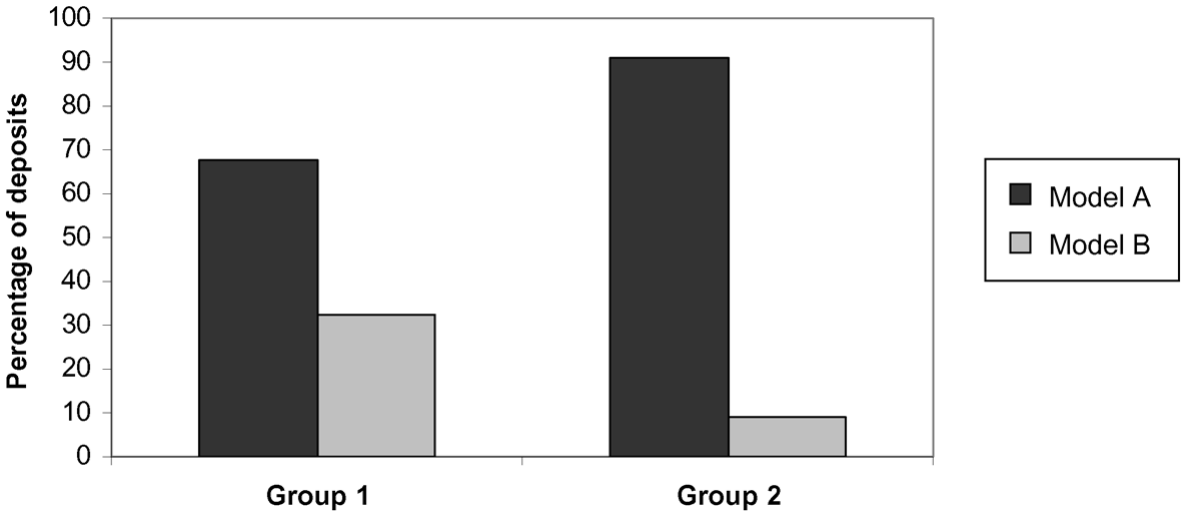
The percentage of deposits into each receptacle by chimpanzees from groups 1 and 2. The receptacles used by models A and B were counterbalanced between groups: the method used by model A in Group 1 was the method used by model B in Group 2, and *vice versa.*

Results showed that new learners differentiated between models in the manner predicted. Chimpanzees deposited significantly more tokens into the receptacle used by model A in both groups than that used by model B. This was true both for a pooled group-level analysis of all deposits (Fisher's-Exact Test comparing Group 1 vs. Group 2 deposits in both receptacles, *P*<0.0001, one-tailed; Fig. 3) and when tested by individual (Wilcoxon signed-ranks test comparing the number of deposits by each chimpanzees into the receptacle used by model A and model B of their respective groups; N-ties = 8, *T* = 3.5, *P*<0.05, one-tailed).

We found no significant difference in the proximity of observers to each trained model during the 10-day observation period (Wilcoxon signed-ranks test comparing the proximity of chimpanzees to models A and B during demonstrations; N-ties = 9, *T* = 21.5, NS). This suggests that observers watched (or at least had an opportunity to watch) both models from an equal distance, and that their subsequent preference for copying model A was not the result of where they happened to be sitting at the time of the demonstrations. In both groups, model A was older, higher-ranking and more experienced than model B.

## Discussion

When given a choice to learn from two conspecific models, chimpanzees showed a significant preference to copy the older, higher ranking individual with a history of success in a similar context (i.e. model A in this study). In human societies, individuals with previously demonstrated skills and knowledge gain “prestige”, such that their actions disproportionately influence the behavior of others [24], [25]. We therefore conclude that in both chimpanzee groups, model A enjoyed a certain level of prestige, which resulted in her behavior being preferentially copied by onlookers.

All successful chimpanzees who used the same method as model A may have been directly influenced by this model during the observation period, but indirect influences may also have occurred during the test trials, if new performers watched each other. Nevertheless, the resulting overall group differences can be traced back to the greater original influence of model A over model B.

Previous discussions of model preference by non-humans have argued that dominant individuals may be copied by subordinates due to fear of aggression, rather than as a result of freely conferred prestige [25]. However, we did not observe aggression related to performance during the current task. Competition over access to the apparatus or food might occur, but chimpanzees do not seem to exert aggressive “peer pressure” related to the solutions that others apply to a problem. In the present study, observers watched both models A and B from approximately the same distance, indicating that the observers' preference for model A did not coincide with fear for A, which would have predicted greater distance from A. Rather, observers appear to have paid selective attention to model A over model B. Model A differed from model B in a combination of characteristics, including social status, reputation for success and age, which are attributes that tend to covary and contribute to prestige in human societies [24], [25]. Further experiments are required to tease apart the relative contributions of these aspects of prestige in chimpanzees. Chance [29] recognized the important role of attention in group social dynamics. Attention also appears to play a central role in the selective transmission of behavior.

In both groups, models A and B demonstrated side by side, often simultaneously, and performed the same number of equally rewarded, hence equally effective, demonstrations. In the wild, such a controlled comparison is unlikely. Older, high-ranking, experienced chimpanzees may be less concerned about competition and scrounging than lower ranking, younger, inexperienced individuals who may subsequently perform on the periphery of the group [30], or at times when other individuals are not paying attention. Additionally, older, high-ranking, experienced chimpanzees may have access to better quality resources and hence their reward pay-offs and efficiency may be greater than others. A preference for copying these “model A” individuals would therefore be beneficial in evolutionary terms, and hence, we predict that in the absence of the stringent controls implemented in this study, the preference to copy prestigious models may be even more pronounced in the natural setting.

Previous studies suggest that chimpanzees are highly conservative with respect to foraging techniques. Once a successful solution has been learned, they are unlikely to switch to an alternative strategy [26], even if it is more efficient than the original method [31], [32]. In the wild, records from over four decades of research at Mahale Mountains National Park in Tanzania have logged 32 innovations in behavior, but most of these have not spread [33]. Other evidence suggests that the majority of chimpanzee innovations are performed by low-ranking individuals, most likely as a means of circumventing competition from dominant group mates [34]. Combining these recent discoveries with our own results suggests that the majority of chimpanzee innovations probably never spread, in part due to the discriminatory preferences shown by chimpanzees for prestigious models. Of course, young, low-ranking individuals may rise in rank and gain prestige so that their innovations may reach the rest of the community with delay. The distribution of chimpanzee cultural behaviors in the wild may therefore be strongly affected by the identity and social characteristics of the original inventors. Further research is needed to explore whether similar processes may be at work in other animal societies, particularly those that are highly structured by social differences.

## Supporting Information

Supporting Information S1 Supporting text and figures giving additional backgroud information about the participants, materials and methods.
(0.10 MB PDF)
