# Supplementary material for: Prestige Affects Cultural Learning in Chimpanzees
Source: PLoS One. 2010 May 19;5(5):e10625. doi: 10.1371/journal.pone.0010625 (PMC2873264; doi:10.1371/journal.pone.0010625)
Supplement: Supporting Information S1 — Supporting text and figures giving additional backgroud information about the participants, materials and methods. (0.10 MB PDF) [file pone.0010625.s001.pdf]

Supporting Information

Prestige affects cultural learning in chimpanzees

Methodology

Participants

This study was conducted at the Yerkes National Primate Research Center Field Station of Emory University. Participants were 14 chimpanzees from two social groups; 8 chimpanzees from Group1 and 6 from Group 2 (see Supporting Table 1). Both groups each house a total of 12 chimpanzees of both sexes and mixed age.

Chimpanzee	Sex	Birth Yr	Group
Georgia	F	1980	1 (model A)
Tara	F	1995	1 (model B)
Anja	F	1980	1
Katie	F	1989	1
Missy	F	1993	1
Peony	F	1968	1
Reinette	F	1987	1
Socrates	M	1987	1
Ericka	F	1973	2 (model A)
Julianne	F	1998	2 (model B)
Amos	M	1981	2
Chip	M	1989	2
Cynthia	F	1980	2
Steward	M	1993	2

Supporting Table 1. Name, sex, age, and group of all chimpanzees who participated in the study.

The chimpanzees live in spacious enclosures with a large outdoor area connected to an indoor housing and research building. This building is comprised of five interconnected bedrooms with nesting sites, swings and toys (outdoor enclosure Group 1 = 711m^2, Group 2 = 528m^2; See Supporting Figure 1).

(a) (b)

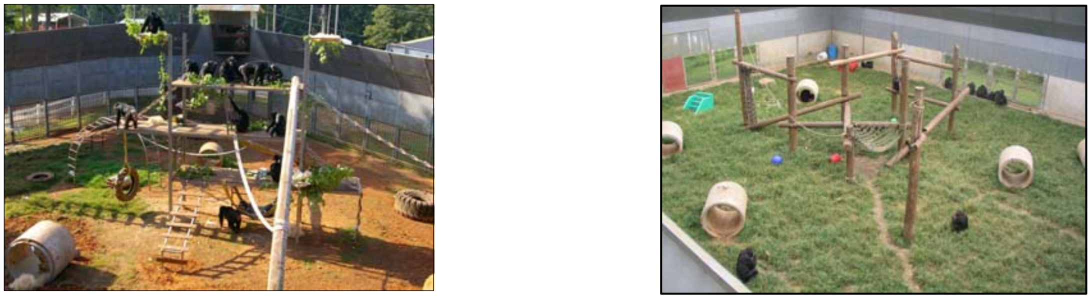

Supporting Figure 1. Yerkes National Primate Research Center: Field Station chimpanzee groups (a) Group 1, (b) Group 2.

Experimental apparatus

In each group, chimpanzees received a token from an experimenter, which could be deposited in to either the striped receptacle (left) or spotted receptacle (right), in order to obtain a food reward from a second experimenter who was standing on an observation tower.

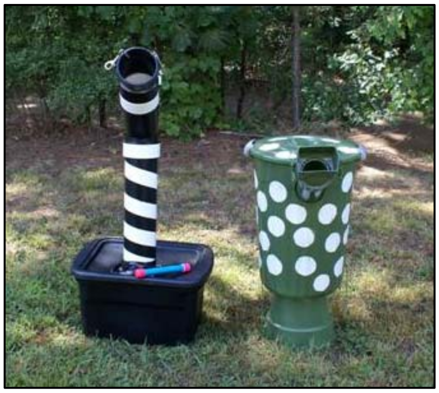

Supporting Figure 2. Photograph of experimental apparatus showing the striped receptacle (left) and spotted receptacle (right). A token is located in the top of the striped receptacle.

Model training

It was important to train each model out of sight of the rest of her group, and hence all other chimpanzees were temporarily locked in their indoor sleeping area during the 10-minute training sessions. In each group, model B was nervous being alone in the outdoor enclosure and so their training sessions were conducted in the presence of one family member who was subsequently excluded from all analyses. Models were deemed to be proficient when they could deposit tokens into the correct receptacle on 10 consecutive occasions on two consecutive days.

Observation period

Once the trained models were proficient, they then participated in the observation period, during which their behaviour could be observed by other chimpanzees in their respective groups. In order to ensure that an equal number of tokens were deposited by both models A and B, tokens were given to them one at a time by an experimenter, who stood exactly between both receptacles outside the enclosure. After successfully completing a demonstration, models received a food reward from a second experimenter who was standing on the observation tower (Fig. 2, main text). All chimpanzees from each group were present during the demonstrations and could potentially observe both models.

Food rewards

Once a token was successfully deposited, a piece of banana was thrown to the model by the second experimenter who was standing on an observation tower that overlooked the enclosure. Delivering food rewards from a tower rather than from the same location as the apparatus prevented the experimenter from unconsciously biasing the chimpanzees' choices and/or drawing their attention away from the task. This procedure was used in both the observation period and the test trials.

Data collection

The experimenter standing between the receptacles at the fence (experimenter 1) used a hand-held digital voice recorder to narrate the proximity of each chimpanzee to the trained models during each demonstration. Proximity was rated as either close (< 10 m from the model) or distant (> 10 m) at the moment that a token was deposited. Video footage of the experiment was recorded from the observation tower by experimenter 2.

Inter-observer reliability

The inter-observer reliability of both experimenters was calculated by comparing the data coded by the first experimenter from the digital voice recorder with the data coded by the second experimenter from the video camera. Both experimenters agreed on the identity, number of deposits and receptacle used by each chimpanzee in 100% of cases. Both experimenters agreed on the proximity of observers to each trained model during demonstrations in 97% of cases. Disagreements resulted from cases in which chimpanzees sat almost exactly on the boundary between the close (<10m) and distant (>10m) proximity categories. All disagreements were resolved by reviewing the video footage.

Critical test trials

After completion of the observation period, all other chimpanzees who approached the experimenter at the fence were given a token which they could deposit into either receptacle to retrieve a reward from the second experimenter who was standing on the observation tower. A total of 40 tokens was made available each day. Sessions continued for 20 minutes, or until all 40 tokens had been used. Experimental sessions were conducted on 3 separate days.

Results

Results confirmed the expectation that observers differentiated between the two available social models, and deposited significantly more tokens in the receptacle used by model A in each group than that used by model B. This was true both for a group-level analysis of all deposits ($P < 0.0001$, one-tailed) and when tested by individual ($P < 0.05$, one-tailed; see Supp. Fig 3).

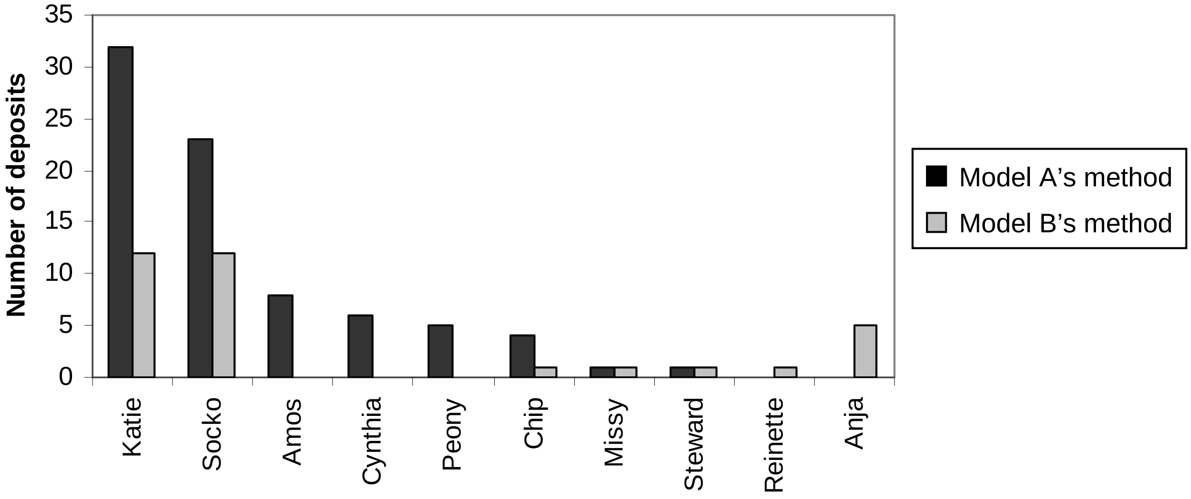

Supporting Figure 3. Total number of deposits into the receptacle used by model A in each group. Data of chimpanzees from both groups have been combined.
